# Evaluating Linkage to Care Among Patients With HIV Viremia in Los Angeles, California

**DOI:** 10.7759/cureus.91612

**Published:** 2025-09-04

**Authors:** Cherie Blair, Hollie M David

**Affiliations:** 1 Department of Medicine, University of California, Los Angeles, USA; 2 Kiran C. Patel College of Osteopathic Medicine, Nova Southeastern University, Davie, USA; 3 Infectious Disease, University of California, Los Angeles, Los Angeles, USA

**Keywords:** healthcare engagement, hiv aids antiretrovial therapy, hiv care, viral load, viral suppression

## Abstract

Multiple barriers hinder retention in the HIV care continuum, including structural, systemic, and individual factors. We conducted a retrospective study to characterize people living with HIV (PLH) in our health system, with detectable HIV-1 viral loads, and identify factors associated with achieving virologic suppression.

We included patients with at least one detectable viral load (HIV RNA >200 copies/mL) and at least one clinical encounter during the study period (from January 1, 2021, to August 31, 2023). We collected demographic, clinical, and healthcare utilization data. Patients were stratified by whether they achieved virologic suppression (HIV RNA <200 copies/mL) following a detectable result. Statistical comparisons were performed to identify significant differences. For the included 216 patients, the median age was 53 years (IQR: 39-61), with 91% (n = 196) male participants. The cohort was racially diverse, with 34% (n = 74) White, 25% (n = 54) Hispanic/Latine, and 20% (n = 43) Black/African American. Most patients (92%) had an active antiretroviral therapy (ART) prescription during the study period.

Of the 117 patients with follow-up encounters, those who achieved virologic suppression were significantly older (median 56 vs. 50 years, p = 0.020) and more likely to initiate or modify ART after their first detectable viral load (74%, n = 39, vs. 50%, n = 32 already on ART, p = 0.009). Hepatitis B/HIV co-infection was observed only among patients with persistent viremia (3.1%, n = 2, vs. 0%, p = 0.002). Patients who achieved suppression underwent more HIV RNA testing (median 3 tests vs. 1 test, p < 0.001), had more total healthcare encounters (median 9 vs. 7, p = 0.003), and had five or more outpatient visits (66%, n = 35, vs. 39%, n = 25, p = 0.027). Conversely, patients with persistent viremia were more likely to be seen in a dedicated HIV clinic (77%, n = 49, vs. 49%, n = 25, p = 0.002).

Older age, ART initiation or modification after detectable HIV viremia, and higher frequency of monitoring and encounters were associated with virologic suppression. Structured follow-up and timely ART interventions may support better outcomes for PLH with detectable viral loads.

## Introduction

In 2023, nearly 40 million people were living with HIV worldwide, with approximately 1.3 million new diagnoses occurring in that year alone, and there were 1,641 new HIV diagnoses in Los Angeles County (LAC) in 2022 [1-2]. The use of antiretroviral therapy (ART) to achieve sustained virologic suppression, defined as HIV quantitative PCR less than 200 copies per milliliter, is critical to prevent transmission and improve health outcomes among people living with HIV (PLH) [3]. In fact, robust clinical data indicates that, among PLH who have achieved sustained virologic suppression with less than 200 copies per milliliter, HIV cannot be transmitted sexually or perinatally [4]. However, suboptimal engagement in the HIV care continuum contributes, in part, to ongoing HIV transmission in LAC, with 76% of newly diagnosed HIV cases linked to care after diagnosis and an estimated 64% achieving virologic suppression in 2023 [5].

Engagement in healthcare is crucial for continued medication adherence required to reach virologic suppression. Barriers to accessing and staying engaged in the HIV care continuum can be partly explained through social epidemiology using the social ecological framework of health [6]. On the systemic level, restrictions on and reduction in health services can exaggerate pre-existing structural and social factors impacting medication availability and adherence [7]. By looking through the lenses of structural, individual and social drivers of health, it may be possible to determine areas that can be improved in engagement and retention in care among PLH. Structural issues, including geographic or economic challenges, and systemic issues, such as access to care and insurance coverage, can contribute to reduced clinical engagement and medication adherence [8-10]. The interface of geographic location with access to health services poses a particular threat to PLH, as access not only to medical care but also trained HIV specialized care is a driver of improved outcomes [11-13]. Individual or social factors like stigma, comorbidities, substance use, lack of social support, financial strain and housing instability may further complicate a patient’s ability to stay engaged in the healthcare system [6,8,14]. Patients who must overcome additional barriers to treatment, such as living farther from healthcare facilities and inadequate health leave to attend healthcare appointments, may be at increased risk of difficulties maintaining engagement in clinical care.

This study aimed to identify demographic and clinical factors associated with viral suppression among patients receiving HIV care in an urban health system, with the intent to inform targeted retention and engagement interventions. This study aimed to identify demographic and clinical factors associated with viral suppression among patients receiving HIV care in an urban health system, with the intent to inform targeted retention and engagement interventions. This analysis was driven by the desire to better understand potential barriers to achieving virologic suppression among PLH receiving HIV care at an academic medical center. Here, we seek to describe PLH who had an encounter at our academic medical center with a detectable viral load (defined as HIV-1 quantitative PCR >200 copies/mL) to evaluate social determinants of health, engagement in care, and clinical characteristics to identify opportunities for interventions that promote engagement and retention across the HIV care continuum. Portions of this article were presented as a meeting abstract at the CHIPTS (Center for HIV Identification, Prevention, and Treatment Services) 2025 HIV Next Generation Conference, January 31, 2025, Los Angeles, California.

## Materials and methods

This is a retrospective case review of de-identified clinical data from PLH who had at least one clinical encounter at our academic medical center with at least one HIV quantitative PCR result >200 (i.e., “detectable” viral load) between January 1, 2021, and August 31, 2023. Eligible patients were those with at least one detectable viral load (HIV RNA >200 copies/mL) and at least one clinical encounter during the study period. We sought to evaluate patient characteristics, proxies for engagement in care, including time between visits, number of missed visits, number of completed visits, and proportion of patients treated at the UCLA CARE (Center for Clinical AIDS Research and Education) Clinic, which is a dedicated HIV clinic, and ART use. For those identified as having a detectable viral load, we extracted data on age, gender, race, ethnicity, zip code, HIV-1 RNA quantitative PCR, current ART prescription, serum creatinine, CD4 count, presence of hepatitis B surface antigen, visit type and whether the patient attended the visit.

Summary statistics were calculated for demographic and clinical laboratory testing results. We calculated the amount of time elapsed (days) between the date of the first detectable viral load test result and (1) first scheduled outpatient clinical visit after the detectable viral load, (2) first completed in-person visit after the detectable viral load, and (3) laboratory result with an HIV viral load <200 (if virologic control was achieved during the study period). The distance between patients’ recorded zip code and the zip code of our academic medical center (90095) was calculated using the zipcodeR package.

All patients who returned to our health system for an appointment after receiving a detectable viral load were sub-categorized into two groups: patients who went on to achieve an undetectable viral load and those who remained detectable throughout the study period. Summary statistics were calculated for demographic and clinical data. The Wilcoxon rank-sum test was applied to continuous variables. Chi-square and Fisher’s exact tests, where appropriate, were used for categorical variables. Analyses were conducted using R version 4.3.3 with RStudio version 2024.12.1+563. R packages dplyr and tidyverse were used for data transformation, and gtsummary was used for statistical analyses.

This study was approved by the UCLA Office of the Human Research Protection Program (24-000969).

## Results

Table 1 summarizes the characteristics of 216 patients with detectable viral loads who completed an encounter at UCLA between January 1, 2021, and August 31, 2023. The median age was 53 years (IQR: 39-61). Approximately 34% of individuals self-identified as White, followed by Latine/Hispanic (25%), Black/African American (20%), and Other race/ethnicity (6.9%). The remaining 14% of the cohort did not have available race/ethnicity data. Most patients were male (91%). Approximately 88% of patients had a negative hepatitis B surface antigen, and 1.4% had hepatitis B/HIV co-infection. The initial detectable viral load count had a median of 1,244 copies per milliliter (IQR: 408-20,061). The majority (92%) of patients had an active prescription for ART during the study period, and 39% of all patients had received an ART prescription before the time of the first detectable viral load. Most participants (87%) had a CD4 count greater than 250 cells/mm³. Most patients (83%) had a creatinine level less than 1.3 mg/dL. Insurance coverage type varied across patients, with 53% having commercial insurance, 25% on Medicare, 9.3% on Medi-Cal, and 12% with unknown/no insurance. Outpatient visits were the most common (95%), while emergency department and inpatient encounters were rare (1.4% and 3.7%, respectively). Among outpatient visits, nearly half of patients (49%) had five or more visits, while 16% had no outpatient visits within the past year. Missed visit patterns showed that 39% of patients had five or more missed appointments. The median distance traveled to receive care was 10 miles (IQR: 6-17).

**Table 1:** Demographic, clinical, and care engagement characteristics of people living with HIV and with at least one HIV quantitative PCR >200 copies/mL during the study period, from January 2021 to August 2023 (N = 216) ART = antiretroviral therapy ^a^Median (IQR: Q1-Q3)

Patient characteristic	n (%)
Age^a^	53 (IQR: 39-61)
Race/ethnicity	
Black/African American	43 (20.0%)
Latine/Hispanic (all races)	54 (25.0%)
Other	15 (6.9%)
Unknown	30 (14.0%)
White	74 (34.0%)
Male gender	196 (91.0%)
Hepatitis B surface antigen	
Non-reactive	189 (88.0%)
Reactive	3 (1.4%)
Receiving ART	198 (92.0%)
CD4 count >250 cells/mm^3^	187 (87.0%)
Serum creatinine <1.3 mg/dL	180 (83.0%)
Insurance category	
Commercial	111 (51.0%)
Medi-Cal	19 (8.8%)
Medicare	50 (23.0%)
Other	36 (17.0%)
Visit type	
Emergency department	3 (1.4%)
Inpatient	8 (3.7%)
Outpatient	205 (95.0%)
Number of outpatient visits in the past year	
0	34 (16.0%)
1-2	38 (18.0%)
3-4	39 (18.0%)
5+	105 (49.0%)
Number of missed visits in the past year	
0	40 (19.0%)
1-2	51 (24.0%)
3-4	40 (19.0%)
5+	85 (39.0%)
Distance from the health system (in miles)^a^	10 (IQR: 6-17)

Characteristics of the 117 patients who had a scheduled outpatient encounter with the UCLA Internal Medicine Department following a detectable viral load during the study period stratified by individuals who achieved virologic suppression with an undetectable viral load and those who did not are described in Table 2. Patients who achieved an undetectable viral load tended to be older, with a median age of 56 years (IQR: 44-64), compared to 50 years (IQR: 39-59) among those with a detectable viral load (p = 0.020). Race and gender distributions were similar between groups. Hepatitis B status differed between the two groups, with 3.1% of patients with detectable viremia having hepatitis B/HIV co-infection, while no cases of hepatitis B/HIV co-infection occurred among patients who achieved virologic suppression (p = 0.002). Patients who remained detectable throughout the study had a median initial detectable viral load of 726 copies per milliliter (IQR: 330-3,386) and those who achieved viral suppression had a median initial detectable viral load of 665 copies per milliliter (IQR: 324-4,389). Active ART prescription before the first detectable viral load varied between groups, with 26% of patients who achieved virologic suppression and 50% of participants who remained detectable throughout the study having an active ART regimen before their first detectable viral load (p = 0.009). Other clinical characteristics, such as creatinine levels and insurance type, did not differ between groups. Differences between groups were observed with regard to visit type, with more patients in the detectable viral load group having healthcare visits in a dedicated HIV clinic (77%) compared to those (49%) who had an undetectable viral load (p = 0.003). Additionally, patients who achieved an undetectable viral load tended to have more outpatient encounters, with 66% having five or more visits compared to 39% among those with a detectable viral load (p = 0.027). Missed visit rates and distance traveled to receive care did not differ between groups.

**Table 2:** Demographic, clinical, and care engagement characteristics of patients living with HIV who had at least one HIV quantitative PCR >200 copies/mL, stratified by whether virologic suppression was achieved during the study period, from January 2021 to August 2023 (N = 117) ART = antiretroviral therapy ^a^Median (IQR: Q1-Q3); n (%) ^b^Wilcoxon rank-sum test; Pearson's chi-square test; Fisher's exact test

Characteristic	Did not achieve viral suppression	Achieved viral suppression	p-value^b^	Test statistic
	N = 64^a^	N = 53^a^		
Age^a^	50 (IQR: 39-59)	56 (IQR: 44-64)	0.02	W = 1271.00
Race/ethnicity			0.2	-
Black/African American	16 (25%)	7 (13%)		
Latine/Hispanic (all races)	18 (28%)	13 (25%)		
Other	2 (3.1%)	6 (11%)		
Unknown	8 (13%)	6 (11%)		
White	20 (31%)	21 (40%)		
Male gender	57 (89%)	47 (89%)	>0.9	X^2^ = 0.00
Hepatitis B surface antigen			0.002	-
Non-reactive	50 (78%)	52 (98%)		
Reactive	2 (3.1%)	0 (0%)		
First detectable viral load^a^	726 (IQR: 330-3,386)	665 (IQR: 324-4,389)	>0.9	W = 1690.00
Overall median viral load^a^	701 (IQR: 307-4,132)	140 (IQR: 68-428)	<0.001	W = 2651.00
Receiving ART	61 (95%)	51 (96%)	>0.9	-
Receiving ART, before the initial detectable viral load	32 (50%)	14 (26%)	0.009	X^2^ = 5.81
CD4 count >250 cells/mm³	61 (95%)	45 (85%)	0.064	-
Serum creatinine <1.3 mg/dL	51 (80%)	45 (85%)	0.5	X^2^ = 0.24
Insurance category			0.5	-
Commercial	32 (50%)	24 (45%)		
Medi-Cal	4 (6.3%)	4 (7.5%)		
Medicare	18 (28%)	11 (21%)		
Other	10 (16%)	14 (26%)		
Received care at a dedicated HIV clinic	49 (77%)	26 (49%)	0.003	-
Number of outpatient visits in the past year			0.027	X^2^ = 9.14
0	10 (16%)	6 (11%)		
1-2	13 (20%)	4 (7.5%)		
3-4	16 (25%)	8 (15%)		
5+	25 (39%)	35 (66%)		
Number of missed visits in the past year			0.3	X^2^ = 3.57
0	12 (194.7%)	6 (11%)		
1-2	10 (16%)	15 (28%)		
3-4	14 (22%)	9 (17%)		
5+	28 (44%)	23 (43%)		
Distance from the health system (in miles)^a^	9 (IQR: 7-21)	11 (IQR: 5-17)	>0.9	W = 1697.5

Table 3 describes encounters and healthcare interactions of 117 patients who achieved viral suppression compared to those who did not. Patients who had a detectable viral load throughout the study period had a median of one HIV quantitative PCR (IQR: 1-2), whereas those who achieved an undetectable viral load had a median of three tests (IQR: 3-6; p<0.001). Patients with a detectable viral load completed a median of seven encounters (IQR: 3-10), whereas those who achieved suppression completed a median of nine encounters (IQR: 6-13; p = 0.003) during the study period. The number of missed in-person encounters was not different between groups, with a median of 6 (IQR: 3-10) among those with detectable viral loads and 5 (IQR: 3-9) among those who achieved virologic suppression (p = 0.9). Among patients with a detectable viral load, 56% successfully completed a follow-up encounter after an initial detectable viral load compared to 72% of those who achieved virologic suppression, though this difference did not reach statistical significance (p = 0.084).

**Table 3:** Care engagement and viral load outcomes by viral suppression status among people living with HIV with initial detectable HIV-1 RNA (>200 copies/mL), who had one healthcare encounter scheduled, from January 2021 to August 2023 (N = 117) ^a^Median (IQR: Q1-Q3); n (%) ^b^Wilcoxon rank-sum test; Pearson's chi-square test

Patient characteristic	Did not achieve viral suppression	Achieved viral suppression	p-value^b^	Test statistic
	N = 64^a^	N = 53^a^		
Viral load tests completed^a^	1.00 (IQR: 1.00-2.00)	3.00 (IQR: 3.00-6.00)	<0.001	W = 3074.5
Proportion of viral load test result >200 copies/mL^a^	1.00 (IQR: 1.00-1.00)	0.33 (IQR: 0.33 - 0.50)	<0.001	W = 197.5
Completed in-person encounters^a^	7 (IQR: 3-10)	9 (IQR: 6-13)	0.003	W = 2241
Missed in-person encounters^a^	6 (IQR: 3-10)	5 (IQR: 3-9)	0.9	W = 1666.5
Completed 1 or more encounters, after viral load result >200 copies/mL	36 (56%)	38 (72%)	0.084	-

The timeline between follow-up HIV quantitative PCR and encounters among the 111 participants with at least one completed encounter after a detectable viral load is described in Table 4. The time interval between a detectable viral load and any subsequent healthcare encounter (e.g., telephone appointment, nurse visit, laboratory visit) was similar between groups, with a median of 28 days (IQR: 10-57) for those with persistent HIV viremia and 26 days (IQR: 12-53) for those who achieved virologic suppression (p = 0.8). The time between a detectable viral load and next completed in-person encounter was also not different, with a median of 48 days (IQR: 19-144) for those with detectable viremia and 33 days (IQR: 15-81) for those who achieved viral suppression (p = 0.11). For patients who achieved viral suppression, the median time from a detectable to an undetectable viral load was 90 days (IQR: 36-196).

**Table 4:** Time between detectable HIV viremia and key clinical events, by viral suppression status among people living with HIV with initial detectable HIV-1 RNA (>200 copies/mL), who had completed one healthcare encounter between January 2021 and August 2023 (N = 111) ^a^Median (IQR: Q1-Q3) ^b^Wilcoxon rank-sum test

Patient characteristic	Did not achieve viral suppression	Achieved viral suppression	p-value^b^	Test statistic
	N = 59^a^	N = 52^a^		
Days between viral load test result >200 copies/mL and next scheduled encounter	28 (IQR: 10-57)	26 (IQR: 12-53)	0.8	W = 1484.5
Days between viral load test result >200 copies/mL and next completed encounter	48 (IQR: 19-144)	33 (IQR: 15-81)	0.11	W = 1266.0
Days between viral load test result >200 copies/mL and viral load test result <200 copies/mL	NA	90 (IQR: 36-196)		NA

## Discussion

Our study evaluates important factors associated with virologic suppression among PLH who received HIV care at an academic medical center in a major metropolitan city. This study serves to describe characteristics of PLH to better understand factors that may pose potential barriers to engagement in the HIV care continuum. Information gained from this study can potentially be used to promote retention in care and improve health outcomes among PLH.

Our analysis revealed that patients who achieved viral suppression tended to be older than those with a detectable viral load, which is consistent with the prior literature demonstrating established links between age and medication adherence [15]. Older patients might have more experience navigating the healthcare system as well as a longer duration of living with an HIV diagnosis, which may result in a greater likelihood of sustained ART adherence and contribute to improved virologic outcomes and engagement in the HIV care continuum [16]. While first detectable viral loads did not differ between those who achieved viral suppression and those who did not, the patients who achieved viral suppression during the study did have lower median viral loads during the study period. These findings further reinforce the importance of viral suppression, as degree of HIV viremia results in systemic inflammation that has been linked to the development of chronic comorbidities, such as cardiovascular disease and stroke [17].

Notably, the location of the clinic visit was different between patients who achieved viral suppression and those who did not. Patients with a detectable viral load were more likely to receive care in a dedicated HIV clinic. Furthermore, patients with detectable viral loads who did not achieve viral suppression were more likely to have had a prescription for ART before the time of their initial detectable viral load. This finding is likely multifactorial. Most PLH in this health system receive HIV care at a stand-alone, dedicated HIV clinic with care delivered by HIV specialists. As this clinic is a designated Ryan White clinic with medical care coordination, social work, and case management resources, including providing care to uninsured and underinsured patients, it is possible that patients receiving care at the dedicated HIV clinic may be more vulnerable to barriers that prevent engagement and retention in care as well as adherence to ART [18,19]. Furthermore, patients receiving care at dedicated HIV clinics may be more likely to be highly treatment-experienced with a higher prevalence of multidrug resistance, potentially posing a barrier to achieving viral suppression [12]. Interestingly, while missed visit rates and travel distances to care facilities did not differ between those who achieved viral suppression and those who did not, the number of viral load tests performed was notably higher among patients who achieved viral suppression. Additionally, those who achieved viral suppression had a shorter interval between a detectable viral load and their next completed in-person visit. This suggests that healthcare engagement beyond structural metrics, potentially including unmeasured factors such as medication adherence, mental health, social support, or housing stability, may play a critical role in achieving suppression. Collectively, these findings suggest that individuals who achieved viral suppression had more frequent contact with the healthcare system and increased healthcare engagement, underscoring the importance of outreach and interventions that improve retention in the HIV care continuum [20].

There are also limitations present in this study. This observational retrospective case review was conducted at a single academic medical center located in Los Angeles, California. Due to the observational nature of the study, we are unable to make direct causal inferences based on our results. Furthermore, as this analysis utilized electronic health data, additional information that may influence virologic suppression and engagement in care, such as ART adherence or transportation, was not captured. This study is limited by the absence of longitudinal follow-up data on post-suppression durability and maintenance of viral suppression. Additionally, our dataset only captures ART prescription dates within our hospital system and does not include systematic information on medication adherence, prior ART exposure at external sites, or HIV drug resistance profiles, which may influence virologic outcomes. Information on ART duration prior to the viral load measurement, adherence, and additional co-infections (e.g., hepatitis C, tuberculosis) was not consistently available in the medical record. Another key limitation of this study is the sample size and selection. The findings may not be generalizable to other health systems, such as rural healthcare settings, telehealth treatment centers, or healthcare systems without a dedicated HIV clinic. Due to the small sample size, we implemented descriptive and exploratory statistical methods to identify basic differences within the cohort, rather than more complex regression methods. Finally, the study did not account for potential confounders such as housing stability, which may influence ART adherence and virologic outcomes.

Overall, our findings reinforce the critical role of continuous engagement in HIV care. Patients who remained actively engaged with healthcare services, demonstrated by higher numbers of completed visits, more frequent viral load testing, and shorter follow-up intervals, were more likely to achieve viral suppression. These results highlight the need for ongoing efforts to promote healthcare engagement, reduce barriers to care, and implement targeted interventions for individuals who may have difficulties achieving virologic suppression. Future research should focus on identifying specific strategies to improve retention in care and optimize health outcomes among PLH.

## Conclusions

Continued engagement in the HIV care continuum is critical for PLH to promote sexual health, overall well-being, and prevent comorbidities. In this study, among PLH who received HIV services at an academic medical center in Los Angeles and had at least one visit with a detectable viral load, we found that patients who achieved viral suppression had more frequent interactions with healthcare services. Patients who had an undetectable viral load had completed more viral load tests, completed more in-person visits, and had a smaller percentage of missed visits after having a detectable viral load. Patients who achieved viral suppression also had shorter follow-up time between a detectable viral load and the next completed in-person visit. Collectively, these findings highlight the importance of continued outreach and support in promoting engagement along the HIV care continuum.
